# SUMOylation: Interplaying with the plant disease triangle

**DOI:** 10.1371/journal.ppat.1013327

**Published:** 2025-07-10

**Authors:** Chi Li, Chengwei Yang, Jianbin Lai

**Affiliations:** Guangdong Provincial Key Laboratory of Biotechnology for Plant Development, School of Life Sciences, South China Normal University, Guangzhou, China; State Key Laboratory of Plant Environmental Resilience, College of Biological Sciences, China Agricultural University, CHINA

Plants are constantly faced with a wide range of pathogens, such as bacteria, fungi, oomycetes, and viruses, leading to significant agricultural losses. The occurrence of efficient pathogen infection depends on the plant disease triangle, which comprises a susceptible host, a virulent pathogen, and a favorable environment. In the wake of severe climate changes, environmental factors are increasingly becoming pivotal in regulating plant-pathogen interactions [1]. During periods of environmental change, post-translational modification (PTM) plays a crucial role in enabling rapid responses within organisms. SUMOylation is an important PTM that involves the transfer of the small ubiquitin-like modifier (SUMO) onto target proteins to regulate their structure and function in eukaryotic cells. SUMOylation has been documented to play a crucial role in both biotic and abiotic stress responses in plants, but the function of this PTM in the interplay between environmental factors and pathogen infection remains ambiguous [2]. Recently, several studies have established a potential link between SUMOylation and the plant disease triangle, thus we aim to summarize these advancements and discuss the model for this interplay.

The development of plant pathogen fungi and their infection are dependent on the presence of suitable environmental conditions. Several previous studies have indicated that SUMOylation in *Magnaporthe oryzae* is crucial for its growth and infection [3,4]. The SUMOylation in this fungus is upregulated by various environmental factors, such as osmotic, alkaline, and oxidative stresses, and is involved in the tolerance to these treatments [3]. Interestingly, two ubiquitination components are required for the upregulation of SUMOylation in *M. oryzae* under heat stress [5], suggesting the crosstalk between ubiquitination and SUMOylation. SUMOylation in *Botrytis cinerea* also plays a regulatory role in the survival of this pathogen to environmental stresses. SUMOylation targets the heat shock protein BcSsb to regulate microtubule stability at low temperatures, as well as the ubiquitin ligase BcRad18 to modulate cell cycle components in oxidative DNA damage responses [6]. This evidence elucidates the molecular mechanism behind the efficient infection of *B. cinerea* at low temperatures. Additionally, another recent work has demonstrated that the SUMOylation status controls the nucleus and cytoplasmic distribution of the *Verticillium dahliae* enolase (VdEno), regulating its role between a glycolytic enzyme and a transcription repressor for controlling effector expression. Interestingly, SUMOylation of VdEno also contributes to cold tolerance of *V. dahliae*, establishing a connection among metabolism, infection ability, and abiotic stress responses [7]. Therefore, SUMOylation contributes to the facilitation of multiple plant pathogen fungi in adaption to diverse environmental conditions as they invade plants.

Unlike fungi, the SUMOylation of proteins from pathogenic bacteria and viruses must take place within plant cells. For instance, NIb, the RNA-dependent RNA polymerase of Turnip mosaic virus, undergoes SUMOylation which regulates its nuclear-cytoplasmic shuttling in plant cells for virus infection [8,9]. Interestingly, NIb suppresses SUMOylation and phosphorylation of NPR1, a key regulator of plant resistance, within the plant nucleus [10]; NIb also acts as a SUMOylation decoy to inhibit RNA quality control in plant cells [11,12]. However, to date, there is no definitive evidence linking abiotic stresses to alterations in the SUMOylation of viral proteins within plant cells, thus it is essential to explore this potential connection in future studies. Recently, it has been demonstrated that at least 16 effectors from *Pseudomonas syringae* are substrates for SUMOylation. It has been observed that under high temperatures, the SUMOylation of two effectors, HopB1 and HopG1, is upregulated in plant cells. The SUMOylation of HopB1 attenuates its cleavage of the plant receptor kinase BAK1, leading to the downregulation of plant cell death. On the other hand, SUMOylation is essential for the function of HopG1 in mitochondria, resulting in ROS accumulation and suppression of jasmonic acid signaling [13]. Thus, dynamic SUMOylation regulates the functions of bacteria effectors differently in response to heat stress; it is plausible that the SUMOylation of specific bacterial effectors may either enhance or inhibit infection. A recent work has demonstrated that MoHTR1, an *M. oryzae* effector, undergoes SUMOylation in rice cells, which is crucial for its localization and stability [14]. However, the potential modulation of fungal effector SUMOylation by abiotic stresses in plant cells remains to be investigated.

In plant cells, SUMOylation is significantly induced by various stresses, but there are limited studies that have established the role of SUMOylation in the interplay between abiotic and biotic stress responses in plants. A previous study has indicated that the mutant of the *SIZ1* gene, which encodes the Arabidopsis SUMO E3 ligase, exhibits SNC1-dependent autoimmunity and enhanced disease resistance at both normal and elevated ambient temperatures [15]. Therefore, SIZ1 functions as a negative regulator of the SNC1-dependent immune response at high temperatures via SUMOylation of the ubiquitin ligase COP1 and the transcriptional co-repressor TPR1, mediating the trade-off between plant immunity and development [16,17]. Because SNC1 is an immune receptor linking to salicylic acid pathway mediated by another SUMOylation substrate NPR1 [18], supporting a general function of SUMOylation in this immunity network. Another study in Arabidopsis has revealed that elevated temperatures influence the expression of genes, including those associated with plant immunity, through chromatin-associated SUMOylation [19]. Furthermore, a number of members of the WRKY and ERF transcription factor families for plant immune responses, were predicted to be targeted to SUMO-associated genomic elements [19], providing important resources for studying SUMOylation in the interplay between immunity and heat responses. Recently, a study demonstrated that the cell wall damage triggers the SUMOylation of Propeps, the precursors of plant elicitor peptides (Peps), to produce active Peps for facilitating plant tolerance [20], suggesting the potential role of this PTM in peptide signaling integral to biotic and abiotic stress tolerance.

Emerging data indicate that temperature fluctuations primarily influence SUMOylation patterns, suggesting that the impact of heat or cold stress on plant immunity and pathogen infection may be mediated, at least in part, by differential SUMOylation of both host and pathogen proteins. In response to various types of stresses, the specificity of SUMOylation is meticulously regulated. In Arabidopsis, SUMO1/2 and SUMO3 play distinct roles in abiotic and biotic stress responses [21], with proteomic studies revealing different SUMOylation profiles under various forms of stresses [22]. Notably, SIZ1 serves as the primary E3 ligase associated with stress responses in Arabidopsis, and its unique domains contribute to differential responses [23]. Given the presence of many de-SUMOylation enzymes in plants, the removal of SUMO from specific substrates may represent a crucial strategy [24]. Consequently, understanding the regulatory mechanisms of SUMOylation specificity on host factors and pathogen effectors in plant cells, as well as on pathogenic proteins in fungal cells, in response to diverse stresses will be an important area for future research.

**Fig 1:**
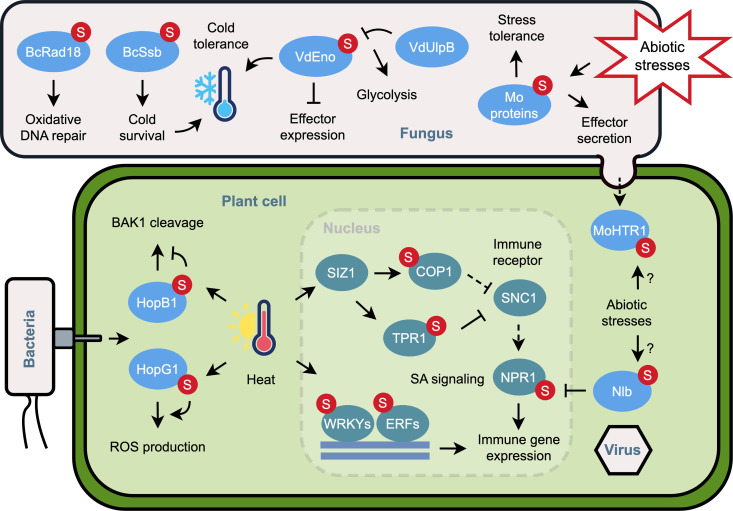
The interplay between protein SUMOylation and the plant disease triangle. The function of SUMOylation, which is associated with environmental factors, in the regulation of pathogenic proteins in fungal cells, effectors in plant cells, and endogenous immunity proteins in plant cells are shown. Plant proteins are represented in dark green, while pathogen proteins are represented in light blue. “S” indicates SUMO; “Mo” indicates proteins from *Magnaporthe oryzae*; “Vd” indicates proteins from *Verticillium dahliae*; “Bc” indicates proteins from *Botrytis cinerea*. Fungal proteins: UlpB (ubiquitin-like protein-specific protease B); Eno (Enolase); Ssb (HSP70 protein); Rad18 (Radiation sensitive 18); HTR1 (Host transcription reprogramming 1). Bacterial effectors: HopB1 and HopG1 (*Pseudomonas syringae*). Virus protein: NIb (Nuclear inclusion B from turnip mosaic virus). Plant proteins: SIZ1 (SAP and MIZ1 domain-containing ligase1, SUMO E3 ligase); COP1 (Constitutive photomorphogenic 1); SNC1 (Suppressor of *npr1-1*, constitutive 1); NPR1 (Nonexpresser of PR genes 1); TPR1 (Topless-related 1); BAK1 (BRI1-associated receptor kinase 1); WRKYs (WRKY DNA-binding proteins); ERFs (Ethylene response factors).

In summary, SUMOylation, which is influenced by environmental factors, may regulate pathogenic proteins in fungal cells, effectors in plant cells, and endogenous immunity proteins in plant cells, interplaying with the plant disease triangle in different ways (Fig 1). The temperature-induced SUMOylation regulates both pathogen infection and plant immunity, possibly as a result of their co-evolution. Therefore, the SUMOylation of specific substrates in fungal and plant cells may have beneficial or detrimental effects on pathogen infection. Additionally, the employment of proteomic approaches will be essential for identifying SUMOylation substrates associated with pathogenesis and immunity in different abiotic situations. Furthermore, an intriguing area of research would involve analyzing the SUMOylation of fungal effectors in both pathogen and plant cells, as well as exploring the interplay between SUMOylated pathogenic and endogenous proteins in plant cells. The interplay between SUMOylation and other PTMs will also represent a compelling area of exploration in this context. Finally, the genetic modification of SUMOylation machinery and substrate sites may be employed to improve crop productivity under various stress conditions [25]. Collectively, these investigations on the plant disease triangle will provide valuable insights into utilizing SUMOylation as a target for enhancing global stress resistance across different crops.
